# Appropriateness of Extremity Magnetic Resonance Imaging Examinations in an Academic Emergency Department Observation Unit

**DOI:** 10.5811/westjem.2018.3.35463

**Published:** 2018-04-05

**Authors:** McKinley Glover, Ravi V. Gottumukkala, Yadiel Sanchez, Brian J. Yun, Theodore I. Benzer, Benjamin A. White, Anand M. Prabhakar, Ali S. Raja

**Affiliations:** * Massachusetts General Hospital, Center for Research in Emergency Department Operations (CREDO), Department of Emergency Medicine, Boston, Massachusetts; † Massachusetts General Physicians Organization, Boston, Massachusetts; ‡ Massachusetts General Hospital, Department of Radiology, Boston, Massachusetts; § Harvard Medical School, Boston, Massachusetts; ¶ Massachusetts General Hospital, Department of Emergency Medicine, Boston, Massachusetts; || Massachusetts General Hospital, Department of Radiology, Division of Emergency Imaging, Boston, Massachusetts

## Abstract

**Introduction:**

Emergency departments (ED) and hospitals face increasing challenges related to capacity, throughput, and stewardship of limited resources while maintaining high quality. Appropriate utilization of extremity magnetic resonance imaging (MRI) examinations within the emergency setting is not well known. Therefore, this study aimed to determine indications for and appropriateness of MRI of the extremities for musculoskeletal conditions in the ED observation unit (EDOU).

**Methods:**

We conducted this institutional review board-approved, retrospective study in a large, quaternary care academic center and Level I trauma center. An institutional database was queried retrospectively to identify all adult patients undergoing an extremity MRI while in the EDOU during the two-year study period from October 2013 through September 2015. We compared clinical history with the American College of Radiology (ACR) Appropriateness Criteria® for musculoskeletal indications. The primary outcome was appropriateness of musculoskeletal MRI exams of the extremities; examinations with an ACR Criteria score of seven or higher were deemed appropriate. Secondary measures included MRI utilization and imaging findings.

**Results:**

During the study period, 22,713 patients were evaluated in the EDOU. Of those patients, 4,409 had at least one MRI performed, and 88 MRIs met inclusion criteria as musculoskeletal extremity examinations (2% of all patients undergoing an MRI exam in the EDOU during the study period). The most common exams were foot (27, 31%); knee (26, 30%); leg/femur (10, 11%); and shoulder (10, 11%). The most common indications were suspected infection (42, 48%) and acute trauma (23, 26%). Fifty-six percent of exams were performed with intravenous contrast; and 83% (73) of all MRIs were deemed appropriate based on ACR Criteria. The most common reason for inappropriate imaging was lack of performance of radiographs prior to MRI.

**Conclusion:**

The majority of musculoskeletal extremity MRI examinations performed in the EDOU were appropriate based on ACR Appropriateness Criteria. However, the optimal timing and most-appropriate site for performance of many clinically appropriate musculoskeletal extremity MRIs performed in the EDOU remains unclear. Potential deferral to the outpatient setting may be a preferred population health management strategy.

## INTRODUCTION

Access to timely healthcare in the United States remains a challenge for many individuals.^1^ One potential downstream result of decreased access to primary and ambulatory care is increased utilization of emergency departments (ED).^2^ Population health management efforts are thus increasingly focused on EDs, with programs aimed at reducing unnecessary ED visits and optimizing appropriate site of care, including the use of ED observation units (EDOU), mobile observation units, and intensive home health programs.^3^–^5^

EDOUs were developed to optimize care for patients who need further evaluation and management but who do not meet criteria for discharge or inpatient admission.^6^ EDOUs have demonstrated benefits in terms of clinical workflow and cost-effectiveness.^7^ However, while EDOUs may provide for a more appropriate and less costly site of care for non-acute patients, they may inadvertently encourage performance of diagnostic workups that may be better suited for outpatient evaluation.

As advanced diagnostic imaging (ADI) has become a critical component of optimal healthcare delivery in the emergency setting, the growth in utilization of advanced imaging has far outpaced trends in general ED use. For example, the use of computed tomography and magnetic resonance imaging (MRI) in the emergency setting increased three-fold over a 10-year period ending in 2007, despite the lack of a commensurate increase in the rate of life-threatening conditions.^8^ Improving appropriate use of ADI is imperative within a healthcare landscape that is increasingly focused on healthcare cost and quality. To that end, the implementation of clinical decision-support (CDS) tools has been demonstrated as highly valuable in improving appropriate ADI use. 9–11 However, while CDS systems for imaging utilization currently focus on appropriateness, they may not adequately provide guidance on appropriate timing (e.g. acuity) and setting of imaging (e.g., inpatient, outpatient, emergent).

Given that a large proportion of ED visits are due to musculoskeletal complaints, MRI is potentially an important diagnostic tool in the emergent setting. The superior ability of MRI to delineate soft tissue injury and bone marrow edema is important in characterizing many musculoskeletal conditions.^12^,^13^ However, as healthcare organizations face increased challenges related to capacity, throughput and appropriate site of care, stewardship of limited and high-cost resources while ensuring excellent clinical outcomes is paramount. Thus, the goal of this study was to assess appropriateness of musculoskeletal extremity MRI examinations in an EDOU at a large academic medical center, based on relevant American College of Radiology (ACR) Appropriateness Criteria® (AC).

## METHODS

### Human Subjects Compliance

This retrospective, Health Insurance Portability and Accountability Act–compliant study was approved by the institutional review board, including waiver of patient consent.

**Population Health Research Capsule**

What do we already know about this issue?

Availability and utilization of magnetic resonance imaging (MRI) in emergency departments has significantly increased; while clinical appropriateness of these studies is not well understood.

What was the research question?

To assess clinical appropriateness of extremity MRI exams performed in an emergency department (ED) observation unit, based on American College of Radiology Appropriateness Criteria.

What was the major finding of the study?

Majority of extremity MRIs performed in the ED observation unit were appropriate based on ACR criteria; questions remain about optimal timing and site of imaging.

How does this improve population health?

Consideration of timing and site of imaging when assessing imaging appropriateness in emergent settings may improve efficiency without compromising care quality

### Study Site

We performed the study at a 999-bed, quaternary care academic center and Level I trauma center. Approximately 111,000 ED visits occur at the institution annually, and approximately 105,000 diagnostic imaging studies are performed and interpreted in the ED radiology division annually. Approximately 10% of ED visits result in further evaluation within the EDOU.

### Collection of Patient Data

We queried an institutional database to identify all adult patients evaluated in the EDOU who underwent an MRI of the extremity (Table 1) while in the EDOU during the study period of October 1, 2013, through September 30, 2015. Patients undergoing MRI in the ED prior to admission to the EDOU were excluded. However, we included patients undergoing MRIs that were ordered while the patient was in ED status, but were performed while the patient was in the EDOU. Patients undergoing MRI of the spine (cervical, thoracic, lumbar, sacrum), pelvis and hip were excluded. For included patients, we also queried the institutional database to determine patient demographic information including age and sex.

Chart review of clinical documented findings within the electronic medical record (EMR) (Partners Healthcare Longitudinal Medical Record, Boston, MA) was performed through use of a data abstraction form designed to capture the following data elements: (1) clinical indication for MRI; (2) appropriateness score of the MRI based on relevant appropriateness criteria; (3) whether surgery was performed, based on review of operative reports; (4) imaging finding categories; and (5) whether subspecialty consultation was performed in the ED, based on documented separate clinical notes from consultants. Chart review was performed by a radiology resident (RG) and radiology fellow (MG). Conflicting data was adjudicated through consensus.

### Outcome Measures

The primary outcome measure was appropriateness of musculoskeletal MRI exams of the extremities, based on relevant ACR AC.^14^ The ACR AC represent an expert panel’s summation of the currently available evidence into a comprehensive set of evidence-based imaging guidelines. The guidelines provide appropriateness scores of various imaging or treatment options for common clinical scenarios. Scores are represented on an ordinal scale from 1 to 9, with 1, 2, and 3 categorized as “usually not appropriate” (i.e., the risks of doing the procedure likely outweigh the benefits); 4, 5, and 6 as “may be appropriate” (i.e., the risk and benefit balance is equivocal); and 7, 8, and 9 as “usually appropriate” (i.e., the benefits of the procedure likely outweigh the risks).

The ACR AC were used retrospectively for this study as they were not part of a clinical CDS system available to physicians at the time of order entry. In cases where a plain radiograph was the most appropriate first exam prior to MRI, the MRI was considered the appropriate second exam only if the radiograph was performed during the ED visit or within seven days prior to the ED visit. We characterized studies dichotomously as “appropriate” for ACR AC scores from 7–9 and “not appropriate” for ACR AC scores of less than seven, a methodology that has been used previously.^15^ For MRI studies categorized as appropriate by this criterion, we then determined if the selected study was the most appropriate option or whether an alternative study with a higher ACR AC score could have been performed.

Secondary outcome measures included data elements within the data abstraction form, which were described in the previous section.

### Statistical Analyses

Data were imported into Stata 14 (StataCorp, College Station, TX) for further analysis. We used summary statistics to describe the distribution of MR examination by extremity, the distribution of indications for MRI exams, and the additional outcome measures discussed above.

## RESULTS

A total of 22,713 patients were evaluated in the EDOU during the study period. Of those patients, 4,409 had at least one MRI performed, and 88 met inclusion criteria for having a musculoskeletal extremity MRI examination, representing 2.0% of all patients undergoing an MRI exam in the EDOU. Forty-eight (55%) extremity MRI exams were ordered while the patient was still in the ED, and 40 (45%) were ordered while the patient was in the EDOU. The mean age of patients included in the cohort was 60 years (standard deviation: 18.2, range 20–99 years); 55% were women.

### Frequency and Distribution of MRI Examinations and Indications

**Figure 1:**
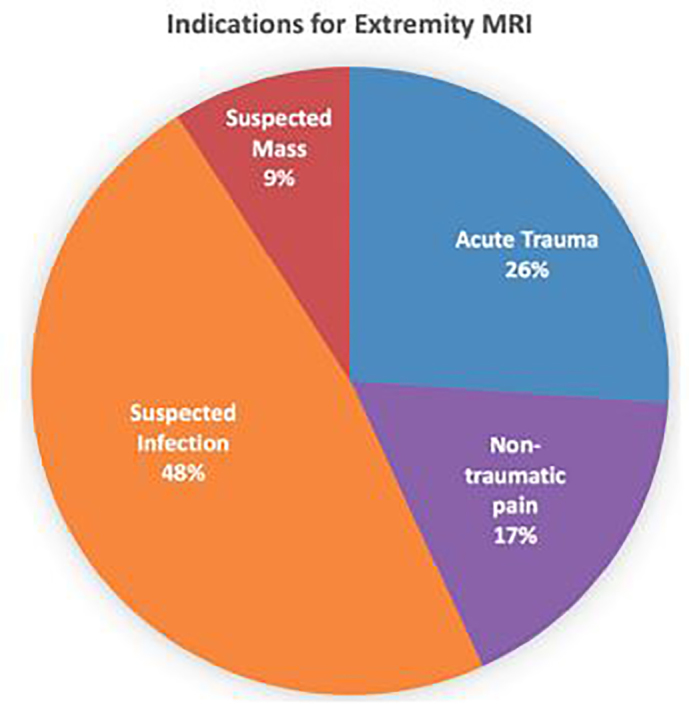
Distribution of indications for musculoskeletal extremity magnetic resonance imaging (MRI) in the emergency department observation unit.

MRI examinations were of the lower extremity in 70 patients (80%) and upper extremity in 18 patients (20%). The most common exams were of the foot (27/88; 31%), knee (26/88; 30%), shoulder (10/88; 11%) and leg (10/88; 11%). Thirty-nine (44%) of the exams were performed with intravenous (IV) gadolinium. The most commons indications were suspected infection (42/88; 48%) and acute trauma (23/88; 26%). MRI examination types and indications are detailed in Figure 1 and Table 1, respectively.

### Appropriateness

**Figure 2:**
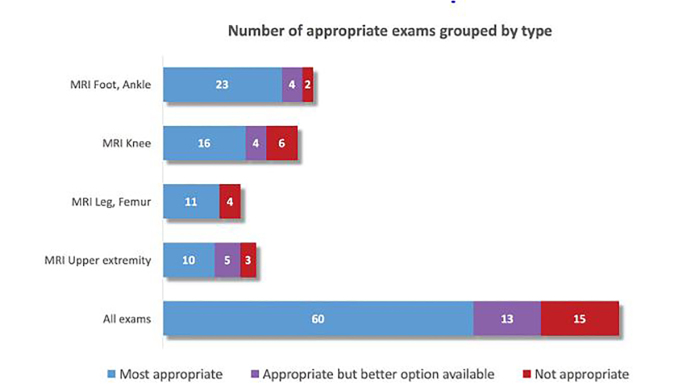
Distribution of magnetic resonance imaging (MRI) appropriateness by body part.

Of the musculoskeletal extremity MRI exams performed, 73 (83%) were deemed appropriate (ACR AC score 7–9). Of exams that were appropriate, 60 (68% of total exams) were the most appropriate option according to the ACR AC. Of the 13 appropriate exams that were not the most appropriate exam, the most common reason was the absence of IV gadolinium, where the exam with the highest ACR AC score would have been an MRI with and without gadolinium. None of these patients had a clear contraindication to the use of gadolinium (allergy or renal dysfunction) based on chart review. In 10 cases (11%), the radiology report for the initial plain radiograph recommended an MRI for further evaluation, and all of the subsequently performed MRIs were appropriate by ACR AC. In 15 of the exams designated as not appropriate, the reason for this designation was the lack of a plain radiograph performed within seven days prior to the MRI study. By strict interpretation of the ACR AC, the fact that the MRI was the first exam performed in these instances led the appropriateness score to be 1 (“usually not appropriate”). The distribution of most appropriate, appropriate, and not appropriate exams, grouped by body part, is depicted in Figure 2.

### Imaging Findings and Additional Outcomes

The most common MRI findings were ligamentous injury (33/88; 38%), joint effusion (14/88; 16%), fluid collection (12/88; 14%), fracture (11, 13%), and osteomyelitis (10/88; 11%), further detailed in Table 2. Nine out of 11 cases of osteomyelitis involved the lower extremity, seven of which involved the foot. The most common consultations obtained while in the EDOU were orthopedic surgery (42/88; 48%), general surgery (5/88; 6%), infectious disease (5/88; 6%), and rheumatology (5/88; 6%), further detailed in Table 3. The most frequent patient disposition following the EDOU visit was home (56/88; 64%), followed by inpatient admission (31/88; 35%) and transfer to a rehabilitation facility (1/88; 1%). Eleven patients (13%) received operative intervention during the same hospital stay.

## DISCUSSION

In this study, we found that musculoskeletal extremity MRI exams represent a very small minority of all MRIs performed in the EDOU. Further, we found that the majority of the musculoskeletal MRIs were appropriate based on ACR AC. Several important conclusions can be drawn. First, our findings demonstrated that although musculoskeletal MRI examinations are not among the commonly ordered MRI exams in the EDOU, clinical providers are typically using a high-cost imaging resource appropriately based on current ACR guidelines. This finding may suggest that within our institution, implementation of CDS systems that require their use prior to ordering may not be of value for this subset of MRI examinations in the EDOU. However, the experience within our institution within this small subset of MRIs performed in the EDOU may not be representative of the larger landscape of MRI use within emergent settings. Multiple prior studies have been shown previously to reduce inappropriate use of advanced diagnostic imaging.^9^,^10^

Second, our study found that nearly half of all patients undergoing musculoskeletal extremity MRI had an orthopedic consultation. Musculoskeletal MRI has roles in evaluation of both traumatic and non-traumatic indications and can be a value-added service in the emergency setting, particularly in guiding management decisions that may alter patient disposition. 12,16,17 These findings suggest that ED providers often collaborate with orthopedic consultants when patients undergo musculoskeletal MRIs. Interestingly, review of clinical notes demonstrated instances in which orthopedic consultants recommended short-term outpatient follow-up and to forego MRI within the ED or EDOU. However, MRI exams were still ultimately performed in these cases, which remained appropriate by ACR AC.

In addition to determining whether imaging, and what type of imaging exam, is appropriate, decisions regarding appropriate timing and location (e.g. acute, emergent, outpatient) are complex. Clinical providers must also account for clinical criteria that may not be included within appropriateness criteria, social situations and/or the ability to obtain appropriate follow-up. However, availability of MRI services in the ED setting may also create incentives to perform exams because of availability.

The development of CDS tools for advanced imaging that incorporate timing of imaging and site of care may be of value in the acute setting. Over time, EDs have become increasingly involved in population health management and primary care.^18^ Deferral of non-urgent (even if technically clinically appropriate) advanced imaging studies to the outpatient setting may help alleviate capacity and resource limitations in the ED. Staying within the EDOU to undergo an MRI and waiting for interpretation may not be in the best interest of the patient if short-term management and disposition will not be altered, given that EDOU stays are often subject to co-insurance.^19^ However, to better optimize the timing and site of care of advanced diagnostic imaging, EDs and hospitals will need to enhance integration with outpatient providers and services to ensure that imaging is well-coordinated and accessible in the ambulatory setting.^20^ Further, within the context of patient experience, the actual and perceived timeliness of results within the ED setting (compared with outpatient follow-up) will also present challenges regarding managing patient expectations while attempting to optimize site of care.^18^,^21^

## LIMITATIONS

This retrospective study had a number of important limitations. The study was conducted in a single large, quaternary care academic medical center serving an urban population and with 24-hour MRI services in the ED, which may limit its generalizability to other sites. In addition, this study did not quantify patients who presented with musculoskeletal complaints and did not undergo an MRI, which limits assessment of overall rates of MR utilization. The determination of exam appropriateness was based on receiving a score of 7, 8 or 9, which has been used in previous studies assessing appropriateness. However, exams with lower appropriateness scores may in fact have been an appropriate examination. Lastly, exams found to be inappropriate may have had recent prior radiographs outside of our healthcare system, but they may not have been available within the EMR or picture archiving and communication system.

## CONCLUSION

The majority of MRI musculoskeletal extremity exams performed in the EDOU were clinically appropriate based on ACR Appropriateness Criteria. However, optimal timing and most- appropriate site for performance of many clinically appropriate musculoskeletal extremity MRIs performed in the EDOU remains unclear.

## Figures and Tables

**Table 1:** Distribution of musculoskeletal extremity MRI exam by body part.

Body part	Number of exams (% of total)
Upper extremity	18 (20%)
Shoulder	10 (11%)
Arm	3 (3%)
Wrist	2 (2%)
Elbow	2 (2%)
Humerus	1 (1%)
Lower extremity	70 (80%)
Foot	27 (31%)
Knee	26 (30%)
Leg	10 (11%)
Femur	5 (6%)
Ankle	2 (2%)
Total	88

*MRI,* magnetic resonance imaging.

**Table 2:** Prevalence of findings on musculoskeletal extremity MRI exams.

Findings	Number of patients (%)
Ligamentous injury	33 (38%)
Joint effusion	14 (16%)
Fluid collection	12 (14%)
Fracture	11 (13%)
Osteomyelitis	10 (11%)
Mass	5 (6%)
Septic arthritis	2 (2%)

*MRI,* magnetic resonance imaging.

**Table 3:** Prevalence of consultations obtained in the ED observation unit for patients undergoing musculoskeletal MRI.

Subspecialty	Number of patients (%)
Orthopedic surgery	42 (48%)
General surgery	5 (6%)
Infectious disease	5 (6%)
Rheumatology	5 (6%)
Podiatry	3 (3%)
Oncology	2 (2%)

*ED,* emergency department; *MRI,* magnetic resonance imaging.
